# Data for functional MRI connectivity in transgender people with gender incongruence and cisgender individuals

**DOI:** 10.1016/j.dib.2020.105691

**Published:** 2020-05-15

**Authors:** Carme Uribe, Carme Junque, Esther Gómez-Gil, Alexandra Abos, Sven C. Mueller, Antonio Guillamon

**Affiliations:** a Medical Psychology Unit, Department of Medicine, Institute of Neuroscience, University of Barcelona, Barcelona; b Centro de Investigación Biomédica en Red sobre Enfermedades Neurodegenerativas (CIBERNED: CB06/05/0018-ISCIII), Barcelona; c Institute of Biomedical Research August Pi i Sunyer (IDIBAPS), Barcelona; d Gender Unit, Hospital Clinic, Barcelona; e Department of Experimental Clinical and Health Psychology, Ghent University, Ghent, Belgium; f Department of Personality, Psychological Assessment and Treatment, University of Deusto, Bilbao, Spain; g Departamento de Psicobiologia, Facultad de Psicologia, Universidad Nacional de Educacion a Distancia, Madrid, Spain

**Keywords:** Functional MRI, gender incongruence, gender identity, graph theory, independent component analysis (ICA), resting-state connectivity, seed-based analysis, transmen, transwomen

## Abstract

We provide T2*-weighted and T1-weighted images acquired on a 3T MRI scanner obtained from 17 transwomen and 29 transmen with gender incongruence; and 22 ciswomen and 19 cismen that identified themselves to the sex assigned at birth. Data from three different techniques that describe global and regional connectivity differences within functional resting-state networks in transwomen and transmen with early-in-life onset gender incongruence are provided: (1) we obtained spatial maps from data-driven independent component analysis using the melodic tool from FSL software; (2) we provide the functional networks interactions of two functional atlases’ seeds from a seed-to-seed approach; (3) and global graph-theoretical metrics such as the smallworld organization, and the segregation and integration properties of the networks. Interpretations of the present dataset can be found in the original article, *doi:10.1016/j.neuroimage.2020.116613*[1]. The original and processed nifti images are available in Mendeley datasets. In addition, correlation matrices for the seed-to-seed and graph-theory analyses as well as the graph-theoretical measures were made available in Matlab files. Finally, we present supplementary information for the original article.

Specifications table

**Table:** 

Subject	Neuroscience, Biological Psychiatry
Specific subject area	Functional MRI connectivity in transgender and cisgender variants
Type of data	Brain MRI images & Correlation matrices Supplementary Tables & Figures
How data were acquired	Magnetic resonance images were acquired with a 3T scanner (MAGNETOM Trio, Siemens, Germany), using an 8-channel head coil. Software: AFNI for imaging basic resting-state pre-processing Python + FSL for ICA-AROMA noise correction FSL for non-parametric statistical imaging analysis Matlab for correlation connectivity matrices
Data format	Raw and analyzed data in nifti (.nii.gz) and Matlab (.mat) formats
Parameters for data collection	T1-weighted images: TR = 2,300 ms, TE = 2.98 ms, TI = 900 ms, flip angle = 9°, matrix size = 256 × 256 mm, 240 slices, resolution = 1 mm isotropic, bandwidth = 240 Hz/pixel, total scan time of 7.48 minutes. T2*-weighted gradient-echo echo planar imaging was acquired in the resting-state: TR = 2,500s, TE = 28ms, flip angle = 80°, matrix size = 256 × 256, 40 slices, resolution = 3 mm isotropic, bandwidth = 2404 Hz/pixel, volumes = 240 (total scan time of 10 minutes), no in-plane GRAPPA, through-plane multiband or any Partial Fourier were used.
Description of data collection	Participants were instructed to keep their eyes closed, not to fall asleep and not to think of anything in particular.
Data source location	University of Barcelona, Barcelona
Data accessibility	Mendeley data repositories: doi:https://doi.org/10.17632/hjmfrv6vmg.2[2], original T1- and T2*-weighted images of both transgender and cisgender individuals. doi:https://doi.org/10.17632/dn82xj4bft.3[3], processed resting-state functional magnetic resonance images (fMRI) of transgender individuals for Independent Component Analysis (ICA, MNI registered and smoothed). doi:https://doi.org/10.17632/zt27ykdrgr.4[4], processed resting-state fMRI of cisgender individuals for ICA (MNI registered and smoothed). doi:https://doi.org/10.17632/rw2yhtpj96.3[5], processed resting-state fMRI of transgender individuals for seed-based and graph-theory analyses (MNI registered, unsmoothed). doi:https://doi.org/10.17632/bgyzz94mz9.3[6], processed resting-state fMRI of cisgender individuals for seed-based and graph-theory analyses (MNI registered, unsmoothed). doi:https://doi.org/10.17632/ts8c7fm8dj.1[7], supplementary data including connectivity matrices, graph-theory measures and ICA spatial maps.
Related research article	Authors: Carme Uribe, Carme Junque, Esther Gómez-Gil, Alexandra Abos, Sven C. Mueller and Antonio Guillamon Title: Brain network interactions in transgender individuals with gender incongruence Journal: Neuroimage 2020 Vol 211, 116613

## Value of the data

• These data provide structural and resting-state functional MRI scans of transgender individuals with gender incongruence. This condition is rare with prevalence rates around 1% in the population [8]. Therefore, study samples tend to be small making this dataset valuable to potentially increase other studies samples.
• These data can be valuable for researchers who aim to explore the underlying neurobiology of different gender variants from cisgender identities to transgenderism by means of MRI.
• These data can be used as an independent sample for studies that aim to characterize and decompose spatial and temporal components into functional group-networks using resting-state imaging in young adults. The use of an independent sample in MRI studies can also be helpful to deal with the *replication crisis*.

## 1 Data

**Fig. 1:**
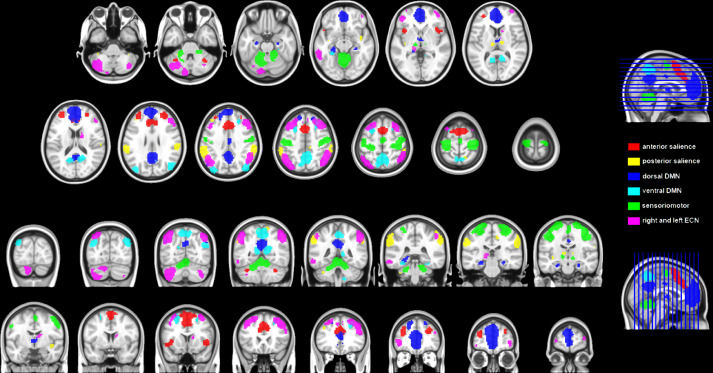
Visual representation of the seeds from the functional Stanford atlas

Data include the original T1-weighted and T2*-weighted images of the 87 participants in the original article [1] that can be found in https://doi.org/10.17632/hjmfrv6vmg.2 [2]. In addition, this dataset includes the 200 functional seeds from the Craddock's atlas [9], and the 56 seeds (from the default mode, salience, executive control and sensorimotor networks) extracted from the Stanford findlab atlas [10] (see Fig. 1). Seeds from these two functional atlases were used in the seed-to-seed and the graph theoretical approches.

The processed resting-state images that were used for the independent component analysis (ICA) approach were published in https://doi.org/10.17632/dn82xj4bft.3 [3] and https://doi.org/10.17632/zt27ykdrgr.4 [4]. After basic pre-processing, images were registered to the standard MNI space and smoothed. The *movement_parameters.txt* file in https://doi.org/10.17632/ts8c7fm8dj.1 [7] contains all motion parameters (max and mean framewise displacement, rotation, translation and correlation between FD and DVARS) reported in Table 1.

**Table 1:** Resting-state group motion parameters

	CM (n=19)	CW (n=22)	TM (n = 29)	TW (n = 17)	H (Kruskal-Wallis test)	p-value
**mean FD**	0.12 (0.05)	0.09 (0.09)	0.11 (0.07)	0.09 (0.08)	3.076	0.380
**maximum FD**	0.45 (0.31)	0.37 (0.29)	0.42 (0.44)	0.53 (0.51)	3.268	0.352
**mean rotation**	0.28 (0.02)	0.02 (0.01)	0.02 (0.01)	0.02 (0.02)	4.160	0.245
**maximum rotation**	0.17 (0.13)	0.12 (0.15)	0.14 (0.18)	0.20 (0.22)	3.243	0.356
**mean translation**	0.06 (0.04)	0.04 (0.05)	0.06 (0.05)	0.04 (0.04)	2.404	0.493
**maximum translation**	0.22 (0.12)	0.18 (0.17)	0.21 (0.24)	0.30 (0.16)	2.957	0.398
correlation FD - DVARS*	0.05 (0.20)	-0.08 (0.17)	-0.14 (0.31)	-0.09 (0.30)	6.778	0.079

⁎ r Pearson correlation between FD and DVARS. Data are medians and interquartile range. Abbreviations: CM, cismen; CW, ciswomen; DVARS, temporal derivative of time courses of root mean square variance over voxels; FD, framewise displacement; TM, transmen; TW, transwomen.

**Fig. 2:**
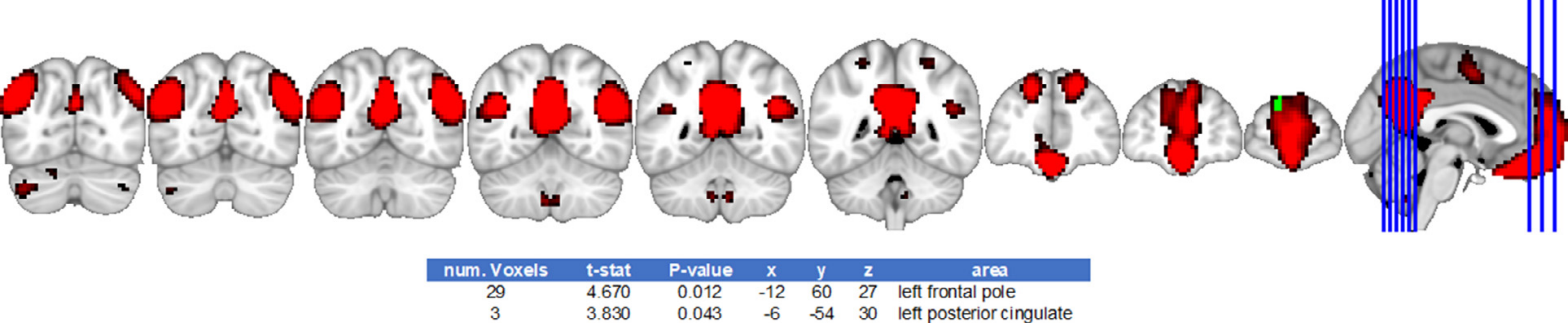
Default mode network differences between cismen > transmen

**Fig. 3:**
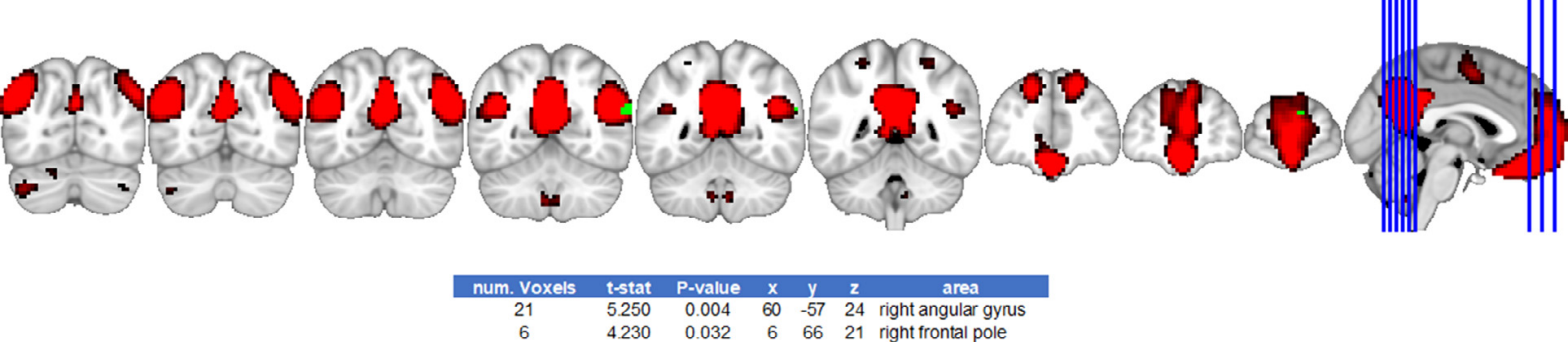
Default mode network differences between ciswomen > transmen

**Fig. 4:**
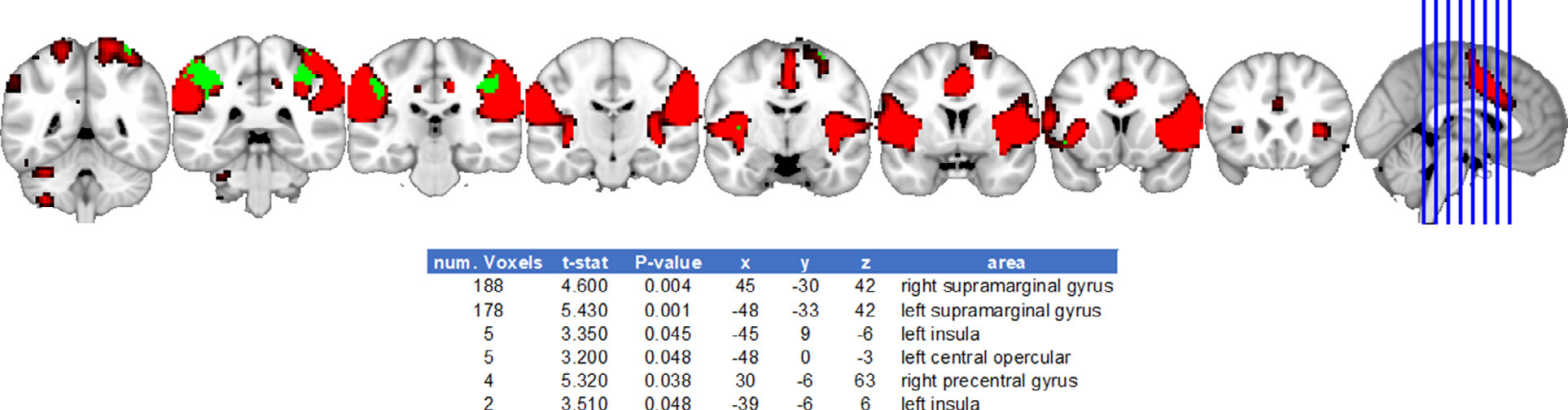
Salience network differences between cismen > transmen

**Fig. 5:**
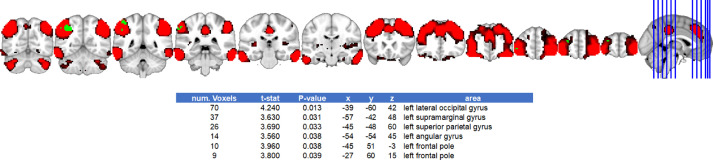
Executive frontoparietal network differences between cismen > transmen

The general linear model (GLM) comparing groups of transgender people with cisgender groups can be found in https://doi.org/10.17632/ts8c7fm8dj.1 [7] and in Fig. 2, Fig. 3–Fig. 4, Fig. 5. GLM regressing out the effects of age and education can be found in the original article [1].

### 1.1 Group comparisons from ICA spatial maps analyses without confounding variables

For the seed-to-seed and graph-theory approaches, unsmoothed images (see https://doi.org/10.17632/rw2yhtpj96.3 [5] and https://doi.org/10.17632/bgyzz94mz9.3 [6]) were used to obtain the correlation connectivity matrices. These matrices can be found in https://doi.org/10.17632/ts8c7fm8dj.1 [7].

Table 2 summarizes the significant test stats and P-values of the edges that differed between cismen > transmen.

**Table 2:** Seed-to-seed functional connectivity analysis between cismen > transmen for the 56 ROIs of the Stanford atlas

	**dDMN** L paracingulate	**LECN** L middle frontal	**RECN** R middle frontal	**dDMN** L lateral occipital	**dDMN** R frontal pole	**anterior salience** R frontal pole	**LECN** R cerebellum	**anterior salience** R cerebellum	**posterior salience** R cerebellum
**anterior salience** L frontal pole		t = 3.198 P = 0.042				t = 3.273 P = 0.040			
**posterior salience** L middle frontal	t = 3.303 P = 0.040	t = 4.223 P = 0.030							t = 3.321 P = 0.040
**anterior salience** L insula	t = 4.736 P = 0.020								
**posterior salience** L supramarginal		t = 3.272 P = 0.040							
**RECN** R frontal pole			t = 3.295 P = 0.050						
**anterior salience** L cingulate	t = 3.170 P = 0.044								
**anterior salience** R frontal pole	t = 3.173 P = 0.042								
**anterior salience** R insula	t = 4.035 P = 0.029								
**posterior salience** R superior parietal					t = 3.063 P = 0.050				
**posterior salience** L insula					t = 3.522 P = 0.045				
**vDMN** R cerebellum							t = 4.111 P = 0.037	t = 4.182 P = 0.036	
**sensoriomotor** L thalamus	t = 3.109 P = 0.045								
**sensoriomotor** L cerebellum				t = 3.256 P = 0.049	t = 3.013 P = 0.050				

dDMN, dorsal default mode network; L, left hemisphere; LECN, left executive control network; R, right hemisphere; RECN, right executive control network; vDMN, ventral default mode network.

P-values are FWE corrected after Montecarlo nonparametric permutation testing from TFNBS.

**Fig. 6:**
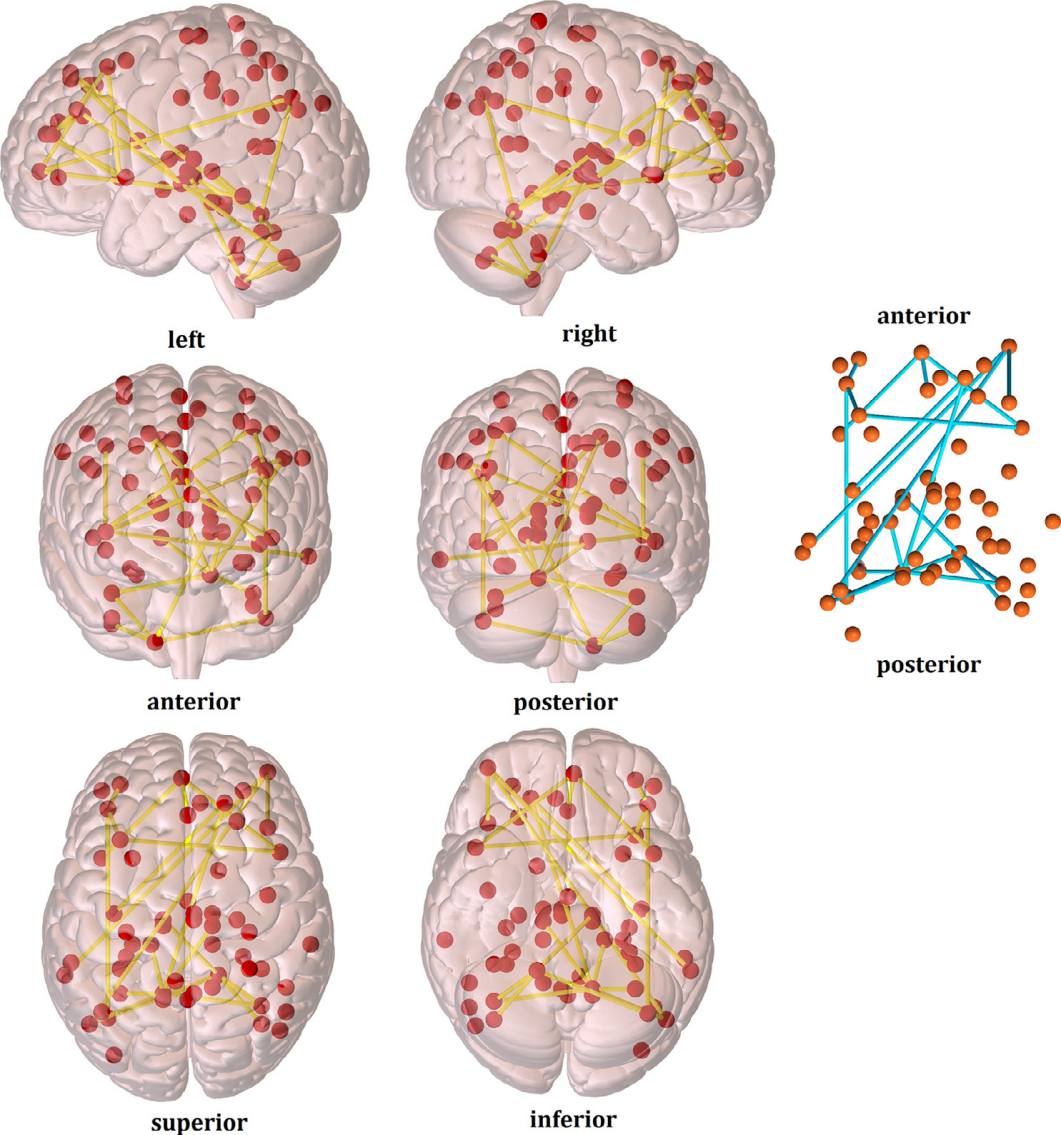
Functional connectivity differences (cismen > transmen) from the seed-to-seed analysis without confounding variables, using the Stanford atlas. *Red dots represent the 56 nodes from the functional template and yellow edges are t tests that reached statistical significance at p-FWE < 0.05 after Montecarlo permutation testing.*

Significant test stats and P-values without any covariates can be found in the *cismen_transmen_transwomen_noncovs_Stanford_atlas_results.mat* file [7] and in Fig. 6.

**Fig. 7:**
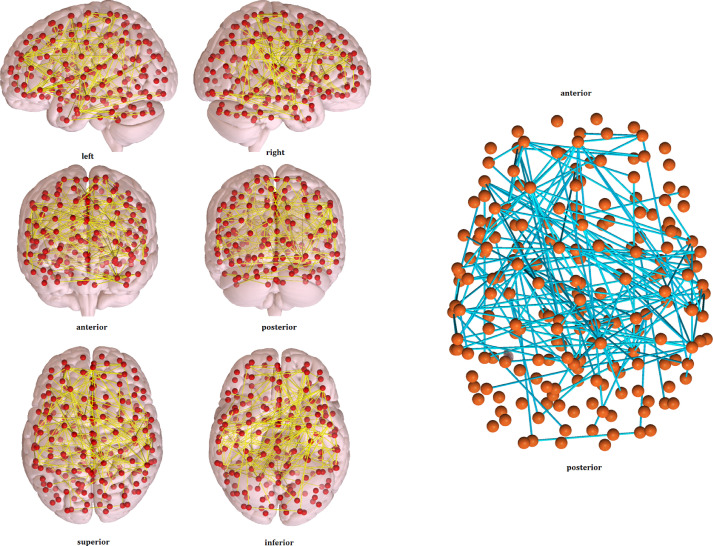
Functional connectivity differences (cismen > transmen) from the seed-to-seed analysis using the Craddock atlas. *Red dots represent the 200 nodes from the functional template and yellow edges are t tests that reached statistical significance at p-FWE < 0.05 after Montecarlo permutation testing.*

Additionally, data for the seed-to-seed group comparisons with the 200 functional ROIs of the Craddock atlas is provided (see the *cismen_transmen_Craddock_atlas results.mat* file [7] and Fig. 7).

Finally, global graph theoretical measures were calculate setting up relative thresholds of sparsity and taking the 100% of the connections (absolute) after deleting connections with negative values. Graph theory measures can be found in the Mendeley repository (doi: https://doi.org/10.17632/ts8c7fm8dj.1 [7]) both for absolute and relative thresholds for the two atlases employed [9,10]. Table 3, Table 4, Table 5 and Table 6 summarizes group means for each graph-theory parameter.

**Table 3:** Graph-theoretical global measures with relative thresholds (Stanford atlas)

	cismen	ciswomen	transmen	transwomen
SW thr 5	3.037 (0.722)	2.496 (0.670)	2.453 (1.353)	2.357 (0.760)
SW thr 7.5	2.494 (0.624)	2.079 (0.434)	2.087 (0.833)	2.098 (0.511)
SW thr 10	2.106 (0.390)	1.887 (0.369)	1.889 (0.577)	1.913 (0.439)
SW thr 12.5	1.881 (0.289)	1.785 (0.315)	1.733 (0.428)	1.814 (0.346)
SW thr 15	1.757 (0.218)	1.636 (0.278)	1.628 (0.341)	1.680 (0.329)
SW thr 17.5	1.622 (0.217)	1.555 (0.252)	1.547 (0.282)	1.562 (0.279)
SW thr 20	1.536 (0.201)	1.468 (0.227)	1.476 (0.231)	1.466 (0.245)
SW thr 22.5	1.449 (0.173)	1.403 (0.198)	1.405 (0.183)	1.387 (0.200)
SW thr 25	1.375 (0.152)	1.350 (0.158)	1.345 (0.167)	1.324 (0.172)
M thr 5	0.557 (0.057)	0.521 (0.072)	0.521 (0.089)	0.494 (0.082)
M thr 7.5	0.493 (0.057)	0.464 (0.062)	0.479 (0.079)	0.454 (0.075)
M thr 10	0.447 (0.046)	0.432 (0.061)	0.441 (0.066)	0.431 (0.075)
M thr 12.5	0.417 (0.054)	0.406 (0.060)	0.411 (0.055)	0.408 (0.070)
M thr 15	0.398 (0.053)	0.387 (0.060)	0.389 (0.053)	0.384 (0.070)
M thr 17.5	0.376 (0.054)	0.367 (0.060)	0.370 (0.052)	0.361 (0.067)
M thr 20	0.363 (0.052)	0.351 (0.061)	0.353 (0.055)	0.345 (0.066)
M thr 22.5	0.347 (0.050)	0.342 (0.059)	0.340 (0.054)	0.327 (0.061)
M thr 25	0.335 (0.050)	0.328 (0.057)	0.328 (0.055)	0.314 (0.061)
CC thr 5	4.391 (0.946)	3.530 (0.907)	3.430 (1.711)	3.206 (1.028)
CC thr 7.5	3.167 (0.791)	2.708 (0.558)	2.706 (1.001)	2.617 (0.688)
CC thr 10	2.566 (0.488)	2.332 (0.474)	2.351 (0.650)	2.297 (0.551)
CC thr 12.5	2.237 (0.379)	2.132 (0.399)	2.089 (0.470)	2.115 (0.442)
CC thr 15	2.054 (0.286)	1.934 (0.356)	1.945 (0.385)	1.951 (0.401)
CC thr 17.5	1.887 (0.276)	1.824 (0.326)	1.824 (0.318)	1.810 (0.346)
CC thr 20	1.790 (0.271)	1.718 (0.292)	1.738 (0.266)	1.698 (0.304)
CC thr 22.5	1.684 (0.237)	1.639 (0.260)	1.652 (0.224)	1.603 (0.257)
CC thr 25	1.601 (0.214)	1.577 (0.211)	1.579 (0.212)	1.528 (0.223)
PL thr 5	1.514 (0.304)	1.464 (0.175)	1.412 (0.327)	1.392 (0.332)
PL thr 7.5	1.284 (0.222)	1.319 (0.102)	1.301 (0.125)	1.246 (0.098)
PL thr 10	1.233 (0.128)	1.240 (0.076)	1.244 (0.130)	1.201 (0.093)
PL thr 12.5	1.195 (0.081)	1.194 (0.065)	1.204 (0.069)	1.164 (0.059)
PL thr 15	1.172 (0.076)	1.182 (0.058)	1.194 (0.054)	1.163 (0.081)
PL thr 17.5	1.164 (0.063)	1.173 (0.049)	1.177 (0.050)	1.158 (0.070)
PL thr 20	1.166 (0.061)	1.169 (0.044)	1.175 (0.059)	1.157 (0.069)
PL thr 22.5	1.162 (0.058)	1.166 (0.044)	1.173 (0.054)	1.155 (0.056)
PL thr 25	1.164 (0.060)	1.167 (0.045)	1.172 (0.054)	1.152 (0.054)

CC, clustering coefficient; M, modularity; PL, path length; SW, small world; thr, threshold.

Data are means and SD.

There were significant differences between cismen > ciswomen in the SW threshold 7.5 (t = 2.1995; P-FWE = 0.028), and between cismen > transwomen in the SW threshold 7.5 (t = 1.968; P-FWE = 0.032), the modularity threshold 5 (t = 2.528; P-FWE = 0.009) and the clustering coefficient threshold 7.5 (t = 2.179; P-FWE = 0.029).

**Table 4:** Graph-theoretical global measures with an absolute threshold (Stanford atlas)

	cismen	ciswomen	transmen	transwomen
smallworld	0.962 (0.048)	0.978 (0.048)	0.968 (0.032)	0.969 (0.039)
modularity	0.218 (0.059)	0.223 (0.066)	0.225 (0.054)	0.208 (0.059)
cluster coefficient	1.134 (0.097)	1.155 (0.102)	1.150 (0.065)	1.136 (0.076)
path length	1.177 (0.049)	1.179 (0.069)	1.187 (0.042)	1.171 (0.060)

Data are means and SD.

There were no significant differences between groups.

**Table 5:** Graph-theoretical global measures with relative thresholds (Craddock atlas)

	cismen	ciswomen	transmen	transwomen
SW thr 5	2.872 (0.572)	2.620 (0.460)	2.471 (0.546)	2.498 (0.475)
SW thr 7.5	2.307 (0.396)	2.116 (0.313)	2.060 (0.373)	2.054 (0.341)
SW thr 10	1.952 (0.296)	1.844 (0.261)	1.806 (0.227)	1.792 (0.264)
SW thr 12.5	1.736 (0.224)	1.652 (0.199)	1.635 (0.233)	1.614 (0.228)
SW thr 15	1.582 (0.181)	1.520 (0.162)	1.518 (0.184)	1.494 (0.195)
SW thr 17.5	1.475 (0.148)	1.419 (0.130)	1.425 (0.151)	1.402 (0.162)
SW thr 20	1.393 (0.127)	1.343 (0.110)	1.351 (0.128)	1.328 (0.143)
SW thr 22.5	1.322 (0.110)	1.282 (0.094)	1.290 (0.109)	1.270 (0.121)
SW thr 25	1.267 (0.096)	1.232 (0.082)	1.241 (0.093)	1.223 (0.108)
M thr 5	0.516 (0.055)	0.497 (0.060)	0.487 (0.056)	0.478 (0.069)
M thr 7.5	0.457 (0.050)	0.437 (0.056)	0.429 (0.053)	0.418 (0.064)
M thr 10	0.413 (0.045)	0.394 (0.056)	0.389 (0.053)	0.381 (0.062)
M thr 12.5	0.380 (0.044)	0.364 (0.054)	0.361 (0.050)	0.352 (0.058)
M thr 15	0.354 (0.044)	0.340 (0.052)	0.337 (0.047)	0.3326 (0.056)
M thr 17.5	0.333 (0.040)	0.321 (0.050)	0.318 (0.046)	0.309 (0.051)
M thr 20	0.316 (0.037)	0.304 (0.049)	0.301 (0.044)	0.290 (0.050)
M thr 22.5	0.302 (0.037)	0.289 (0.048)	0.285 (0.044)	0.277 (0.049)
M thr 25	0.289 (0.036)	0.276 (0.049)	0.274 (0.042)	0.264 (0.047)
CC thr 5	3.548 (0.719)	3.180 (0.620)	2.989 (0.679)	3.013 (0.635)
CC thr 7.5	2.768 (0.486)	2.500 (0.424)	2.422 (0.463)	2.422 (0.467)
CC thr 10	2.312 (0.355)	2.149 (0.348)	2.097 (0.340)	2.088 (0.358)
CC thr 12.5	2.045 (0.270)	1.916 (0.273)	1.887 (0.283)	1.867 (0.3005)
CC thr 15	1.860 (0.221)	1.757 (0.226)	1.747 (0.227)	1.724 (0.265)
CC thr 17.5	1.732 (0.182)	1.638 (0.189)	1.638 (0.189)	1.616 (0.224)
CC thr 20	1.636 (0.158)	1.550 (0.166)	1.553 (0.165)	1.530 (0.200)
CC thr 22.5	1.554 (0.137)	1.479 (0.146)	1.483 (0.142)	1.464 (0.175)
CC thr 25	1.490 (0.120)	1.422 (0.131)	1.427 (1.123)	1.409 (0.159)
PL thr 5	1.235 (0.042)	1.211 (0.059)	1.210 (0.059)	1.202 (0.071)
PL thr 7.5	1.199 (0.036)	1.179 (0.048)	1.175 (0.046)	1.175 (0.065)
PL thr 10	1.185 (0.036)	1.163 (0.044)	1.161 (0.042)	1.162 (0.060)
PL thr 12.5	1.178 (0.033)	1.157 (0.042)	1.154 (0.039)	1.155 (0.054)
PL thr 15	1.175 (0.033)	1.154 (0.041)	1.150 (0.039)	1.151 (0.052)
PL thr 17.5	1.174 (0.032)	1.152 (0.041)	1.149 (0.038)	1.151 (0.051)
PL thr 20	1.174 (0.032)	1.152 (0.041)	1.149 (0.038)	1.151 (0.051)
PL thr 22.5	1.175 (0.032)	1.152 (0.041)	1.149 (0.038)	1.151 (0.051)
PL thr 25	1.176 (0.032)	1.153 (0.042)	1.149 (0.039)	1.151 (0.052)

CC, clustering coefficient; M, modularity; PL, path length; SW, small world; thr, threshold.

Data are means and SD.

There were significant differences between:

- **cismen > ciswomen** in the SW threshold 7.5 (t = 1.704; P-FWE = 0.059), in the CC thresholds 5 (t = 1762; P-FWE = 0.056) and 7.5 (t = 1.858; P-FWE = 0.041), in the PL thresholds 17.5 (t = 1.732; P-FWE = 0.050), 20 (t = 1.761; P-FWE = 0.050), 22.5 (t = 1.785; P-FWE = 0.045) and 25 (t = 1.812; P-FWE = 0.040);

- **cismen > transwomen** in SW thresholds 5 (t = 2.163; P-FWE = 0.022), 7.5 (t = 2.115; P-FWE = 0.025) and 10 (t = 1.740; P-FWE = 0.058); in PL thresholds 5 (t = 1.685; P-FWE = 0.050), 12.5 (t = 1.646; P-FWE = 0.051), 15 (t = 1.756; P-FWE = 0.043), 17.5 (t = 1.723; P-FWE = 0.047), 20 (t = 1.756; P-FWE = 0.043), 22.5 (t = 1.737; P-FWE = 0.046), 25 (t = 1.803; P-FWE = 0.036); in M thresholds 5 (t = 1.930; P-FWE = 0.028), 7.5 (t = 2110; P-FWE = 0.017), 10 (t = 1.760; P-FWE = 0.045), 12.5 (t = 1.625; P-FWE = 0.058), 15 (t = 1.697; P-FWE = 0.053), 20 (t = 1.720; P-FWE = 0.050), 22.5 (t = 1.684; P-FWE = 0.054) and 25 (t = 1.758; P-FWE = 0.045), in CC thresholds 5 (t = 2.406; P-FWE = 0.017), 7.5 (t = 2.256; P-FWE = 0.022), 10 (t = 1.928; P-FWE = 0.047), 12.5 (t = 1.888; P-FWE = 0.051), 20 (t = 1.857; P-FWE = 0.053) and 25 (t = 1.830; P-FWE = 0.056);

**- cismen > trasmen** in the SW threshold 5 (t = 2.731; P-FWE = 0.009), 7.5 (t = 2.763; P-FWE = 0.009), 10 (t = 2.398; P-FWE = 0.014), 12.5 (t = 2.099; P-FWE = 0.029) and 15 (t = 1.790; P-FWE = 0.054), in M thresholds 5 (t = 1.895; P-FWE = 0.032), 7.5 (t = 1.923; P-FWE = 0.032), 10 (t = 1.759; P-FWE = 0.053), in CC thresholds 5 (t = 2.938; P-FWE = 0.007), 7.5 (t = 2.894; P-FWE = 0.007), 10 (t = 2.577; P-FWE = 0.009), 12.5 (t = 2.311; P-FWE = 0.014), 15 (t = 2.069; P-FWE = 0.024), 17.5 (t = 1.955;; P-FWE = 0.036), 20 (t = 1.945; P-FWE = 0.036), 22.5 (t = 1.851; P-FWE = 0.045) and 25 (t = 1.813; P-FWE = 0.049), in PL thresholds 15 (t = 1.676; P-FWE = 0.054), 17.5 (t = 1.725; P-FWE = 0.048), 20 (t = 1.747; P-FWE = 0.047), 22.5 (t = 1.768; P-FWE = 0.044) and 25 (t = 1.807; P-FWE = 0.038).

**Table 6:** Graph-theoretical global measures with an absolute threshold (Craddock atlas)

	cismen	ciswomen	transmen	transwomen
smallworld	0.960 (0.029)	0.972 (0.032)	0.979 (0.034)	0.969 (0.041)
modularity	0.204 (0.032)	0.195 (0.050)	0.197 (0.036)	0.188 (0.045)
cluster coefficient	1.138 (0.039)	1.125 (0.051)	1.131 (0.036)	1.124 (0.058)
path length	1.185 (0.034)	1.158 (0.042)	1.156 (0.040)	1.160 (0.052)

Data are means and SD.

There were significant differences in path length between cismen > ciswomen (t = 2.043; P-FWE = 0.015), between cismen > transwomen (t = 1.817; P-FWE = 0.037), and between cismen > transmen (t = 1.990; P-FWE = 0.018).

## 2 Experimental Design, Materials, and Methods

### 2.1 Participants

Twenty-nine transmen (TM) and 17 transwomen (TW) with gender incongruence according to the ICD-11 with an identification with the other gender; and 22 ciswomen (CW) and 19 cismen (CM) underwent MRI evaluation.

### 2.2 MRI acquisition

T1 and T2*-weighted images were acquired in a SIEMENS MAGNETOM TrioTim syngo MR B19. T1-weighted images were acquired in the sagittal plane using a MPRAGE iPAT GRAPPA (PAT 2), TR = 2,300 ms, TE = 2.98 ms, TI = 900 ms, echo spacing = 7.1 ms, flip angle = 9°, matrix size = 256 × 256 mm, 240 slices, resolution = 1 mm isotropic, bandwidth = 240 Hz/pixel, total scan time of 7.48 minutes. T2*-weighted gradient-echo echo planar imaging was acquired in the resting-state: TR = 2,500s, TE = 28ms, echo spacing = 0.48 ms, flip angle = 80°, matrix size = 256 × 256, 40 slices, resolution = 3 mm isotropic, bandwidth = 2404 Hz/pixel, volumes = 240 (total scan time of 10 minutes), no in-plane GRAPPA, through-plane multiband or any Partial Fourier were used.

### 2.3 MRI preprocessing

Basic functional image preprocessing, using AFNI (http://afni.nimh.nih.gov/afni) tools, included: discarding the first five volumes to allow magnetization stabilization, despiking, motion correction, grand-mean scaling, linear detrending, and temporal filtering (maintaining frequencies above 0.01 Hz).

We selected 56 ROI from a functional template [10]. The downloaded ROI had a resolution of 2 mm in standard MNI space and they were transformed to a slice thickness of 3 mm with the flirt tool from FSL software.

We also used the 200 ROIs of the functional Craddock's atlas [9]. From the MNI coordinates (https://rdrr.io/cran/brainGraph/man/craddock200.html), we created spheres of 5 mm with the FSL software as previously described (http://andysbrainblog.blogspot.com/2013/04/fsl-tutorial-creating-rois-from.html).

### 2.4 Head motion parameters and noise correction

To control for head motion, an exclusion cut-off was established for mean interframe head motion at ≥ 0.3 mm translation or 0.3° rotation; and for maximum interframe head motion at ≥ 1 mm translation or 1° rotation.

To remove the effects of head motion and other non-neural sources of signal variation from the functional data, we used an ICA-based strategy for Automatic Removal of Motion Artifacts (ICA-AROMA) [11]. ICA-AROMA breaks data down via ICA and automatically identifies, which, if any, of these components are related to head motion by using four robust and standardized features (https://github.com/maartenmennes/ICA-AROMA).

As a quality control measure to assess the efficacy of ICA-AROMA in reducing relationship between signal variation and motion, we performed correlations between framewise head displacement and overall signal variation after regressing the ICA-AROMA components.

### 2.5 ICA spatial maps and dual regression

Melodic from FSL v5.0.10 (https://fsl.fmrib.ox.ac.uk/fsl/fslwiki/) was used to obtain temporal-concatenated spatial maps based on an ICA approach. Temporally and spatially coherent patterns of signal variation were extracted from functional images with a predetermined dimensionality of 25. The SN, the DMN and the bilateral ECN were considered to be the networks of interest.

The set of spatial maps from the group-average analysis was used to generate subject-specific versions of the spatial maps, and associated timeseries, using FSL's dual regression. First, for each subject, the group-average set of spatial maps is regressed (as spatial regressors in a multiple regression) into the subject's 4D space-time dataset. This ends in a set of subject-specific timeseries, one per group-level spatial map. Next, those timeseries are regressed (as temporal regressors, again in a multiple regression) into the same 4D dataset, resulting in a set of subject-specific spatial maps, one per group-level spatial map. We then tested for group differences using the FSL permutation-testing tool (5,000 permutations) with threshold-free cluster enhancement (TFCE). A binarized mask for each network was applied.

### 2.6 Intra and internetwork functional connectivity differences

The first eigenvariate of the BOLD signal temporal series was extracted for the ROI form the two atlases with the *fslmeants* command (https://fsl.fmrib.ox.ac.uk/fsl/fslwiki/Fslutils). The connectivity between two ROI was estimated using Pearson's correlation between their time series. Therefore, a 56 × 56 matrix and a second 200 × 200 matrix were obtained for each of the 87 subjects. Seed-to-seed intergroup differences in the strength of the edges were obtained with the threshold-free network-based statistics, TFNBS [12]. This method performs statistical inference on brain graphs and combines network-based statistics [13], frequently used for statistical analysis of brain graphs, and TFCE, a method commonly used in voxel-wise statistical inference [14]. Matlab R2017a (The MathWorks Inc., Natick, MA, USA) was used to perform t test and Montecarlo permutation testing with 1,000 iterations between each of the four groups with and without considering age or education as confounding variables. Reported information survived Bonferroni connectome-wise correction for multiple comparisons at P < 0.05.

### 2.7 Graph-theory measures

A graph-theory approach was applied using the same matrices (56 × 56 × 87 for the Stanford atlas and 200 × 200 × 87 for the Craddock's atlas). Networks were constructed using only positive r values, i.e., setting negative values to 0. We used a sparsity threshold to create a set of undirected graphs (existing number of edges in a graph divided by the number of all possible edges) using the r correlation values as edge weights for each pair of seeds for each subject. Sparsity is a measure of network density that ensures that all subjects’ networks would have the same number of edges to facilitate group comparisons. The range of sparsities was 5 to 25% with incremental steps of 2.5%. These percentages mean that, in the first threshold, 95% of the weakest connections will be deleted, 92.5% for the second and so on. The global graph theory measurements computed were:

The *clustering coefficient,* which quantifies the number of connections that exist between the neighbors of a node as a proportion of the maximum number of possible connections. As a global measurement, it is the average of the clustering coefficient of all nodes. This measurement was normalized by 1,000 random networks (generated by random rewiring of the original network, maintaining degree distribution).

*The characteristic path length* of a node, which is the average of the minimum number of edges that must be traversed to go from this node to any other network node. As a global measurement, it is the average of the characteristic path length of all nodes. This measurement was normalized by 1,000 random networks.

*Modularity,* indicates the degree to which a network can be subdivided into well-delineated modules, each containing several densely interconnected nodes with relatively few connections between nodes in different modules.

*Small world coefficient*, defined as the ratio of the average clustering coefficient to the characteristic path length divided by the ratio of the same measurements obtained from equivalent random networks. Networks that have this small-world property usually have coefficients >1.
